# Assessing the validity of the ICECAP-A capability measure for adults with depression

**DOI:** 10.1186/s12888-017-1211-8

**Published:** 2017-02-02

**Authors:** Paul Mark Mitchell, Hareth Al-Janabi, Sarah Byford, Willem Kuyken, Jeff Richardson, Angelo Iezzi, Joanna Coast

**Affiliations:** 1 School of Social and Community Medicine, University of Bristol, Bristol, UK; 2 The National Institute for Health Research Collaboration for Leadership in Applied Health Research and Care West (NIHR CLAHRC West), University Hospitals Bristol NHS Foundation Trust, Bristol, UK; 3 UK Renal Registry, Southmead Hospital Bristol, Bristol, UK; 4 Health Economics Unit, Institute of Applied Health Research, University of Birmingham, Birmingham, UK; 5 King’s Health Economics, King’s College London, London, UK; 6 Department of Psychiatry, University of Oxford, Oxford, UK; 7 Centre for Health Economics, Monash University, Melbourne, Australia

**Keywords:** Patient reported outcome measures, Quality of life, Health economics

## Abstract

**Background:**

Effectiveness and cost-effectiveness are increasingly important considerations in determining which mental health services are funded. Questions have been raised concerning the validity of generic health status instruments used in economic evaluation for assessing mental health problems such as depression; measuring capability wellbeing offers a possible alternative. The aim of this study is to assess the validity of the ICECAP-A capability instrument for individuals with depression.

**Methods:**

Hypotheses were developed using concept mapping. Validity tests and multivariable regression analysis were applied to data from a cross-sectional dataset to assess the performance of ICECAP-A in individuals who reported having a primary condition of depression. The ICECAP-A was collected alongside instruments used to measure: 1. depression using the depression scale of the Depression, Anxiety and Stress Scale (DASS-D of DASS-21); 2. mental health using the Kessler Psychological Distress Scale (K10); 3. generic health status using a common measure collected for use in economic evaluations, the five level version of EQ-5D (EQ-5D-5L).

**Results:**

Hypothesised associations between the ICECAP-A (items and index scores) and depression constructs were fully supported in statistical tests. In the multivariable analysis, instruments designed specifically to measure depression and mental health explained a greater proportion of the variation in ICECAP-A than the EQ-5D-5L.

**Conclusion:**

The ICECAP-A instrument appears to be suitable for assessing outcome in adults with depression for resource allocation purposes. Further research is required on its responsiveness and use in economic evaluation. Using a capability perspective when assessing cost-effectiveness could potentially re-orientate resource provision across physical and mental health care services.

**Electronic supplementary material:**

The online version of this article (doi:10.1186/s12888-017-1211-8) contains supplementary material, which is available to authorized users.

## Background

For health services to prioritise mental health interventions, both effectiveness and cost-effectiveness are key considerations. Regulatory bodies such as the National Institute for Health and Care Excellence (NICE) in England and Wales have evolved methods for establishing cost-effectiveness across both health and mental health interventions with the use of economic evaluations [1]. The recommended approach in measuring quality of life patient benefits for economic evaluations has been to use generic, health focused patient reported outcome measures like EQ-5D [2, 3]. Once patients have completed such measures and population preferences have been attached to patient reported health states, health related quality of life (HRQoL) scores are used to generate quality-adjusted life years (QALYs), a composite measure accounting for the benefits of improved morbidity and reduced mortality [4].

Depression results in the second largest disease burden in terms of disability-adjusted life years (DALYs) globally (DALYs are a similar, but slightly different measure to QALYs [5]), with the condition also a major determinant in suicide and associated with ischemic heart disease [6]. Therefore, it is crucial for any provision of health care that instruments used in deciding how to allocate resources are able to assess the impact of depression on a person’s quality of life and the improvements from providing treatment and preventing the illness. Although recent findings show HRQoL measures fare better in patients with depression compared to other mental health groups such as patients with bipolar disorder and schizophrenia, there is an acknowledgement of the restricted coverage of themes important to all mental health patients in the most commonly used HRQoL measures: EQ-5D and SF-6D [7].

There are many critiques of QALYs. These include worries about the impact on equity of using QALY maximisation as the objective for economic evaluation [8–10] and concerns about whether QALYs are fully able to capture broader patient value from health care [11–14]. This latter topic has received particular focus in the past decade with unease about a sole reliance of just health related aspects of quality of life, emanating from a number of different research groups [15–17]. Nevertheless, the ability to use QALYs to compare across very different health situations has meant that it has retained its position of prominence, in particular QALYs using EQ-5D [18]. A recent study into the methods for assessing cost-effectiveness by NICE found that the current standard approach leads to a negative impact on QALYs forgone in a number of areas including forgone gains from mental health services [19].

An alternative, relatively new approach for assessing outcomes for individuals has emerged which focuses on people’s capabilities [20]. The capability approach, developed most notably by Nobel prize winning economist Amartya Sen, is primarily concerned with the evaluation of individual advantage based on a person’s ability to achieve ‘functionings’ in life that are valuable to them [20]. Examples of ‘functionings’ in the capability approach range from basic attainments such as nourishment to more complex attainments such as having self-respect. In essence, the capability approach attempts to provide a more encompassing picture of individuals when assessing the impact of policy decisions upon them, than approaches that prefer to focus on “objects of convenience”, such as income when assessing household and national wellbeing [20].

The capability approach has been promoted by bioethicists [21, 22], philosophers [23, 24], and health service researchers [25, 26] who see the approach as a method to assess impacts on an individual in an alternative evaluative space than that of preference-based HRQoL measures used to produce QALYs. This perspective has also proved helpful in conceptualising mental health problems. Hopper [27] found the capability perspective accommodating when trying to determine social recovery for patients with schizophrenia [27]. More recently, other researchers have assessed Community Treatment Orders for patients with mental health disorders (predominantly schizophrenia) through a capability lens by developing a 16 item questionnaire [28] based on a list of ten central human capabilities conceptualised by eminent philosopher Martha Nussbaum [29].

The capability approach has gained recognition by NICE, which has added capability measures to its reference case for conducting economic evaluations where non-health benefits are likely to accrue [1]. Currently, two capability measures are recommended by NICE, an instrument for social care known as Adult Social Care Outcomes Toolkit (ASCOT) [30], and one of a generic family of instruments developed originally from a grant called Investigating Choice Experiences for the Preferences of Older People (ICEpop), that has developed capability (CAP) instruments for Adults (ICECAP-A), Older people (ICECAP-O) and a Supportive Care Measure (ICECAP-SCM) for those in need of palliative care (www.birmingham.ac.uk/icecap), with the earliest developed measure (ICECAP-O) currently recommended. If the same principles of comparability across diseases are used for justifying the need for generic HRQoL instruments when allocating resources across a health service, a similar argument can be made for using the generic ICECAP-A, the only index of capability currently developed that could be applied across a broad range of patient groups from populations of adults aged 18 years and older [31].

As a newly developed measure, evidence for the measurement properties of the ICECAP-A in patient groups is so far limited. It is important that any new outcome measure, including those used in economic evaluation, demonstrates satisfactory measurement properties [32]. One test of whether the measure is valid (i.e. it measures what it purports to measure) is “construct validity”. This involves testing mini-theories that are developed to explain the relationship between the characteristic of interest (in this case, capability) and other relevant characteristics of the individual [33].

The overall aim of this work is to assess the validity of the ICECAP-A measure in a sample of individuals suffering from depression. Validity in this study is assessed in three ways. First, concept mapping, a qualitative construction of pathways or constructs between capability and depression related items is developed based on a synthesis of qualitative research in a mental health population [34]. The concept mapping framework then allows for the formation of hypotheses about where a relationship is expected between capability and depression related items. Second, discriminant validity, the ability of a measure to discriminate between identifiable groups, is tested by using information about the ICECAP-A overall score in relation to clinical cutoffs on two condition-specific questionnaires. Finally, multivariable regression is conducted to compare the explanatory power of mental health items and overall scores attached to the measure of capability (ICECAP-A) and the NICE recommended HRQoL measure for economic evaluation, the EQ-5D-5L [3]. This enables an assessment of how a generic measure of capability compares with the recommended HRQoL measure in economic evaluation. Even though the measures are developed using different underlying constructs, regression analysis provides a means of comparing to current methodology recommended by NICE for economic evaluation. We hypothesise that more capability (through ICECAP-A) than health status (through EQ-5D-5L) will be explained by depression related items, because we expect the items on a capability measure capturing “psychosocial well-being” will have more in common for individuals with depression than the “physical functioning” focused EQ-5D [35]. The three methods of validity assessment use two measures closely related to depression, the depression anxiety and stress scale (DASS-21) [36] and the Kessler (K10) psychological distress scale [37].

## Methods

### Data set and collection

The study uses data from a Multi Instrument Comparison (MIC) cross sectional survey of individuals in eight health categories (http://www.aqol.com.au) conducted between November 2011 and May 2012. The survey was conducted in six countries of which the four English speaking nations (Australia, Canada, United Kingdom and United States) were chosen for the present analyses (as the choice of these countries did not raise issues associated with translation of the measures). As well as a healthy population, seven broad health condition populations were targeted. This study utilises data from individuals who reported depression as their primary condition, as well as the healthy population, from the four English speaking nations [38].

The MIC survey was conducted online with panel members using a global survey company, CINT Pty Ltd. The personal and medical details recorded by the company were used to recruit individuals from the health condition groups. Quota sampling was conducted for health condition groups to reach a total number in each health condition group irrespective of age, sex and education. The survey sought a sample of 150 individuals in each health condition area per country to ensure statistical power. Individuals were asked to complete a relevant disease specific questionnaire (two instruments for those with a diagnosis of depression) to confirm the existence of the illness and to measure its severity. Choice of clinical measures for each health condition was informed by expert opinion of commonly used measures in the source country of the MIC survey (i.e. Australia).

Ethics approval was obtained from Monash University Human Research Ethics Committee (MUHREC Approval CF11/1758: 2011 00074). At the start of the MIC survey, a Participant Information and Consent form was provided. Proceeding with the survey was deemed as provision of consent by the participant.

### Measures

#### Capability measure

##### ICECAP-A

The descriptive system for the ICECAP­-A instrument was developed in the UK using qualitative methods [31]. The five capabilities captured by ICECAP-A are phrased in terms of “being able to be” or “can have” and are *stability* (“settled and secure”), *attachment* (“love, friendship and support”), *autonomy* (“independent”), *achievement* (“achieve and progress”) and *enjoyment* (“enjoyment and pleasure”) (ICECAP-A available from www.birmingham.ac.uk/icecap). These five items make up the battery of questions in the ICECAP-A and they attempt to capture broad concepts related to people’s capability to live a life that they value. The *stability* item concerns informants’ desire for continuity in their lives when it came to friends, work and location. The *attachment* item emphasises how informants placed emphasis on love, support and social contact. The *autonomy* attribute came out of a desire to be one’s own person and not a liability to others. The *achievement* attribute represents how informants placed value on moving forward in life and attaining their goals. Finally, the *enjoyment* attribute tried to capture everyday enjoyment that people want to be able to have in their lives [31]. The attributes aim to capture the capabilities that people value as distinct from the factors that determine capability (e.g. income, health) [31].

Each capability item has four levels of responses. Once a response level for each attribute is selected by the patient, general population values can then be attached to the patient’s current state. The use of population values, as opposed to patient values, is the preferred approach for health guidance bodies such as NICE [1]. The general population approach to valuing states means that all possible individual states across a health service can be, in theory, compared to one another. UK population values have been developed for the ICECAP-A [39]. The measure is anchored at 1 (full capability) and 0 (no capability). Values can range from 0 to 1.

The ICECAP-A has been validated for the general adult UK population [40]. Additionally, a qualitative ‘think-aloud’ investigation has been conducted to assess the ease of interpretation and completion of the attributes of capability captured on the ICECAP-A [41]. Content validity [42] and test-retest reliability of the instrument in the general population have also been examined [43]. Responsiveness of the ICECAP-A has been assessed in an osteoarthritis patient group [44]. As of January 2017, the ICECAP-A was registered for use in 64 studies across 10 countries: Australia, Canada, China, Denmark, Ireland, Mexico, the Netherlands, New Zealand, the UK and the USA. Originally developed in English, studies have or are attempting to translate the measure into seven other languages (Chinese, Dutch, French, German, Spanish, Turkish and Welsh).

#### Condition-specific measures

##### Depression Anxiety and Stress Scale 21 item (DASS-21)

The Depression Anxiety and Stress Scale 21 item (DASS-21) is an abbreviated version of the original DASS 42 item questionnaire [36]. It is a set of three self-report scales (7 items per scale) that was designed to measure the negative emotional states of depression, anxiety and stress over the past week. The DASS-21 has been validated in clinical [45] and non-clinical [46] samples. The depression sub-scale (DASS-D), which is of primary interest in this study, contains seven questions relating to the dimensional syndromes associated with depression: dysphoria (state of unease), hopelessness, devaluation of life, self-depreciation, lack of interest/involvement, anhedonia (inability to experience pleasure) and inertia [36]. Each item is rated on a 4-part Likert scale, with scores ranging from 0 to 3 per item, with higher scores reflecting more severe states. Five severity levels (normal, mild, moderate, severe and very severe) have been developed and those who score more highly display a number of characteristics, such as being self-disparaging; dispirited, gloomy and blue; convinced that life has no meaning; pessimistic about the future; unable to experience enjoyment and satisfaction; unable to become interested or involved; and slow, lacking in initiative [36].

##### Kessler Psychological Distress Scale (K10)

The Kessler Psychological Distress Scale 10 item (K10) was developed for the US National Health Interview Survey to identify people with a serious mental illness [37]. The questionnaire consists of 10 items measuring ‘psycho-social distress’ [37]. There is also a shortened 6 item version (K6) [37]. There are five response levels for each question, with scores ranging from 1 to 5 and higher scores representing higher psychological distress. There are four severity levels on the K10 (well, mild depression and/or anxiety disorder, moderate depression and/or anxiety disorder and severe depression and/or anxiety disorder). While primarily used in non-clinical settings [47, 48], the K10 has also been tested for detecting depression and anxiety disorders in primary care [49].

#### Generic health status measure

##### EQ-5D-5L

The EQ­-5D­-5L is an updated version of the original EuroQol (EQ­-5D) generic measure of health status [2, 50]. The instrument is recognised as one of the most widely used generic measures of health status. It has been translated into 169 different languages. EQ­-5D data are routinely collected in some countries as well as being used to inform healthcare decision-making [51]. The instrument has five dimensions (mobility, self-care, usual activities, pain/discomfort and anxiety/depression). Each dimension has five response levels [3]. Preliminary general population values for the EQ-­5D-­5L have been developed from the three level version for a number of countries [52], with research ongoing for new value sets for the five level version. The measure is anchored at 1 (best health) and 0 (death), with a minimum value of -0·594 for the UK value set. Values thus range from -0·594 to 1. There has been one major study to date assessing the validity of EQ­-5D-­5L compared to the original instrument across a variety of chronic conditions including depression [53].

### Analysis

#### Concept mapping from condition-specific and capability items

Concept mapping is an approach used to organise and analyse qualitative research findings and has been previously applied in mental health research [54]. We built a conceptual framework to establish ‘pathways’ between depression items and capability items and used concept mapping to organise qualitative information into testable quantitative hypotheses based on instrument completion by people with depression. Data collected on capability (using ICECAP-A) and depression (using DASS-D and K10 condition-specific measures) from people reporting depression as their primary condition were then used to test these hypotheses and establish how depression was likely to influence capability.

The pathways between condition-specific items and ICECAP-A items were informed by a systematic review of qualitative research in people with mental health problems [34], to identify how mental health affected quality of life and a person’s capability in people with depression. In their study, Connell and colleagues [34] identified six themes of quality of life that were of importance for adults with mental health problems: well-being and ill-being; control, autonomy and choice; self-perception; belonging; activity; hope and hopelessness [34]. These six themes each comprised 3 to 10 attributes that contributed to these respective aspects of quality of life. For example, the self-perception domain consisted of four related attributes: self-identity/sense of self; self-efficacy; self-esteem; and self-acceptance/self-stigma [34].

To establish whether these intermediary mental health themes identified by Connell et al. [34] related to capability and depression, we used judgement informed by expert opinion (SB, WK). Based on the qualitative findings from the development of the ICECAP-A, three to four key attributes per item were identified from Connell and colleagues’ synthesis and attached to the five capability items by PM, HA and JC (see Table 1) [31]. The items of DASS-D and K10 were also compared to Connell’s 6 themes. PM used these qualitative themes to develop hypotheses about likely relationships between the ICECAP-A and the condition-specific instruments.

**Table 1:** Key concepts underpinning the 5 items of the ICECAP-A measure

Stability	Attachment	Autonomy	Achievement	Enjoyment
Continuity of friends	love	look after oneself	move forward in life	quiet pleasures
Continuity of work	support	independence in decision-making	attain goals	fun
Continuity of location	social contact	privacy	pride	exciting
		identity	recognition and appreciation	

First, the ICECAP-A, DASS-D and K10 items were individually compared to the quality of life themes and attributes of the themes for people with mental health problems identified by Connell and colleagues [34]. Step 1 assigned pathways between attributes from quality of life themes and items on measures that were determined by PM to be related to one another.

Step 2 drew upon the mappings established in Step 1 from Connell’s mental health themes to the three instrument items, by then mapping between the ICECAP-A items with the seven DASS-D items and the ten items on the K10. A pathway between a depression item and a capability item was hypothesised when a depression item and capability item were linked to at least half of intermediaries in common as identified by Connell and colleagues. So, for example, an ICECAP-A item that conceptually mapped onto two of the six themes identified by Connell and colleagues [34], say ‘well-being, ill-being’ and ‘belonging’, would have an expected relationship with a depression item on DASS-D mapped onto three mental health themes, where two of these three themes included the same two themes as mapped onto the ICECAP-A item (80% or 4 of 5 themes in common). A relationship between items on different measures would not hold if the depression item, mapped onto three themes, only had one of two themes in common with the capability item (40% or 2 of 5 themes in common) (detailed explanation in Additional file 1). This process was conducted initially by PM and checked by SB and WK to assess whether the face validity of these relationships would hold in a clinical sample of patients with depression. The associations between all ICECAP-A items and items on the condition-specific questionnaires were assessed using chi-squared tests (categorical variables) to assess these hypothesised pathways.

#### Discriminant validity

Discriminant validity is used to test the ability of the ICECAP-A to differentiate individuals classified with different existing severity levels on the condition-specific questionnaires. It was expected that the ICECAP-A index score would differ significantly between those classified in different depression severity levels on the DASS-D (5 levels) and the K10 (4 levels). The difference in means between severity groups is tested using the Mann-Whitney *U* test.

#### Multivariable regression analysis

Finally multivariable regression analysis, using Ordinary Least Squares (OLS), was used to assess the degree to which overall capability (ICECAP-A) and health utility (EQ-5D-5L) was explained by the items of (i) the DASS-D; (ii) K10 and (iii) K6 (derived from the K10 responses). It was expected that, if capability is better at capturing the depression-related attributes of adults with depression than the EQ-5D-5L, the resultant R^2^ would be higher for the overall capability models than for the health status models. The R^2^ coefficient is a statistic that shows, in this case, how well depression-related items explain capability or health utility on a 0-1 scale, where 1 is capability fully explained by depression-related items and 0 represents no relationship between depression items and capability.

## Results

In total, 617 individuals who reported depression as their primary condition were included in the analysis. Table 2 shows the distribution of the ICECAP-A responses across the sample. As a comparator, responses from a representative healthy population sample across the same four countries (*n* = 965) are also included. In terms of percentages, a higher proportion of the depression sample recorded responses on the lowest two levels of capability across all five ICECAP-A items and a higher proportion of the healthy population recorded responses in the top two levels.

**Table 2:** ICECAP-A responses from sample of individuals with depression (*n* = 617) and healthy population (*n* = 965)

	Depression		Healthy	
1. Feeling settled and secure (STABILITY)	n	%	n	%
I am able to feel settled and secure in all areas of my life	35	5.7	365	37.8
I am able to feel settled and secure in many areas of my life	195	31.6	474	49.1
I am able to feel settled and secure in a few areas of my life	271	43.9	112	11.6
I am unable to feel settled and secure in any areas of my life	116	18.8	14	1.5
2. Love, friendship and support (ATTACHMENT)				
I can have a lot of love, friendship and support	138	22.4	552	57.2
I can have quite a lot of love, friendship and support	214	34.7	317	32.8
I can have a little love, friendship and support	225	36.5	92	9.5
I cannot have any love, friendship and support	40	6.5	4	0.4
3. Being independent (AUTONOMY)				
I am able to be completely independent	244	39.5	692	71.7
I am able to be independent in many things	249	40.4	244	25.3
I am able to be independent in a few things	100	16.2	24	2.5
I am unable to be at all independent	24	3.9	5	0.5
4. Achievement and progress (ACHIEVEMENT)				
I can achieve and progress in all aspects of my life	73	11.8	437	45.3
I can achieve and progress in many aspects of my life	219	35.5	449	46.5
I can achieve and progress in a few aspects of my life	251	40.7	75	7.8
I cannot achieve and progress in any aspects of my life	74	12.0	4	0.4
5. Enjoyment and pleasure (ENJOYMENT)				
I can have a lot of enjoyment and pleasure	62	10.0	519	53.8
I can have quite a lot of enjoyment and pleasure	183	29.7	374	38.8
I can have a little enjoyment and pleasure	334	54.1	68	7.0
I cannot have any enjoyment and pleasure	38	6.2	4	0.4

For the original ICECAP-A questionnaire, visit www.birmingham.ac.uk/icecap

Table 3 presents socio-demographic information for this sample. The average ICECAP-A score (0.637) is considerably lower for people with depression in this sample than that from a general UK adult population (≈0.83) [39, 40], and also lower than the “healthy population” sample collected simultaneously in the same dataset across the four countries (≈0.89) [55].

**Table 3:** Socio-demographic information for the sample of respondents reporting depression

Key variables	Sample Size	(%)
Sex		
Female	416	67.4
Male	201	32.6
Age		
18-24	64	10.4
25-34	158	25.6
35-44	142	23.0
45-54	148	24.0
55-64	85	13.8
65+	20	3.2
Highest Education		
High School	215	34.8
Diploma/certificate/trade	214	34.7
University	188	30.5
Living with partner/spouse		
Yes	341	55.3
No	276	44.7
Country of Residence		
Australia	146	23.7
Canada	145	23.5
United Kingdom	158	25.6
United States	168	27.2

Total population = 617; ICECAP-A average = 0.637 (0.620,0.654);

EQ-5D-5L average = 0.593(0.572,0.614)

### Concept mapping

**Fig. 1:**
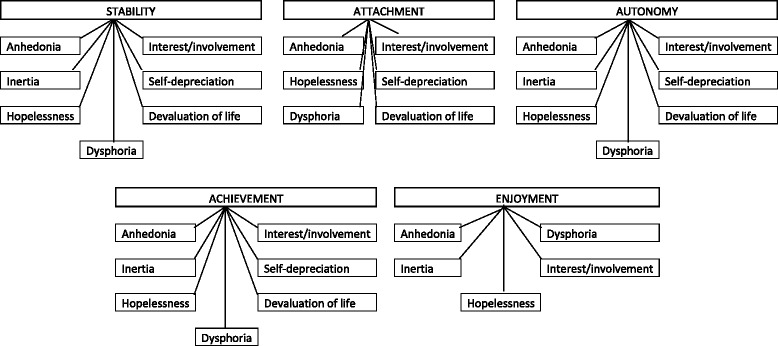
Concept mapping from DASS-21 depression Scale onto ICECAP-A capability attributes

In Fig. 1, based on the qualitative mapping exercise (see Additional file 1 for full details), the expected pathways between the five ICECAP-A items and the 7-item depression scale on the DASS-21 questionnaire are shown. Out of the 35 potential mappings from the depression scale onto the ICECAP-A attributes (7x5 = 35), 32 were expected to produce significant relationships (Table 4). A similar process was conducted for the K10 and ICECAP-A attributes. From the 50 potential relationships, 40 were predicted through the mapping process to produce significant relationships (Table 5). All 85 potential relationships were tested using chi-squared tests, with all 85 relationships resulting in significant results reporting a p-value of less than 0.05.

**Table 4:** Concept mapping DASS-D and ICECAP-A item relationships by mental health themes (%)

	Stability	Attachment	Autonomy	Achievement	Enjoyment
Anhedonia	**0.89**	**0.57**	**0.67**	**0.91**	**0.57**
Inertia	**0.75**	0.33	**0.75**	**0.80**	**0.67**
Hopelessness	**0.80**	**0.50**	**0.80**	**1.00**	**0.50**
Dysphoria	**0.89**	**0.57**	**0.89**	**0.91**	**0.57**
Lack of interest/involvement	**0.86**	**0.80**	**0.57**	**0.67**	**0.80**
Self-depreciation	**0.57**	**0.80**	**0.88**	**0.67**	0.40
Devaluation of life	**0.50**	**0.67**	**0.75**	**0.80**	0.33

Relationships in bold where items between two measures have at least half of Connell’s mental health themes in common

**Table 5:** Concept mapping K10 and ICECAP-A relationships by mental health themes (%)

	Stability	Attachment	Autonomy	Achievement	Enjoyment
K1	**0.67**	**0.50**	**0.67**	**0.50**	**0.50**
K2	0.33	**0.50**	**0.67**	**0.50**	**0.50**
K3	0.33	**0.50**	**0.67**	**0.50**	**0.50**
K4	**0.80**	**0.50**	**0.80**	**1.00**	**0.50**
K5	0.40	**0.67**	0.40	0.29	**0.67**
K6	0.40	**0.67**	0.40	0.29	**0.67**
K7	**0.80**	**0.50**	**0.80**	**1.00**	**0.50**
K8	**0.75**	0.40	**0.75**	**0.80**	**0.67**
K9	**1.00**	**0.67**	**0.75**	**0.80**	**0.67**
K10	**0.57**	**0.80**	**0.86**	**0.67**	0.40

K10, Kessler Psychological Distress Scale 10 item; k1, tired for no good reason; k2, feel nervous;

k3, nervous so that nothing could calm you down; k4, hopeless; k5, restless or fidgety;

k6, restless that you could sit still; k7, feel depressed; k8, everything was an effort;

k9, so sad that nothing could cheer you up; k10, worthless. Relationships in bold where items between two measures have at least half of Connell’s mental health themes in common

### Discriminant validity

**Fig. 2:**
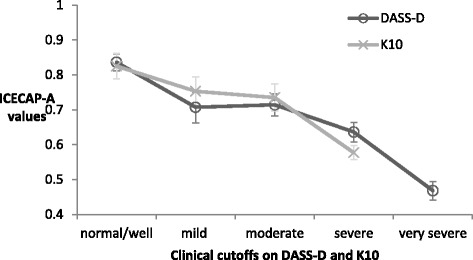
ICECAP-A scores and clinical cutoffs on condition-specific instruments. ICECAP-A: capability instrument on a scale of 1(full capability) to 0 (no capability); DASS-D, depression scale on the Depression, Anxiety and Stress Scale 21 item measure. K10, Kessler psychological distress scale. DASS-D clinical cutoffs: normal (*n* = 112); mild (*n* = 71); moderate (*n* = 100); severe (*n* = 129); very severe (*n* = 205). K10 clinical cutoffs: well (*n* = 44); mild (*n* = 68); moderate (*n* = 88); severe (*n* = 417). The error bars represent 95% confidence intervals around the mean

Figure 2 displays the ICECAP-A scores based on clinical cut-offs using the Depression scale on DASS-21 [36] and the K10 scale [37]. ICECAP-A mean values are able to discriminate between severe and less severe states (DASS-D very severe (0.47) and severe (0.64)), as well as the normal/well group (DASS-D 0.84) from those classified in the mild group (DASS-D 0.71). There is not a clear distinction of the ICECAP-A values between the mild and moderate groups on both the DASS-D (both 0.71) and the K10 (mild 0.75, moderate 0.74).

In Tables 6 and 7, the models of capability and health status from items on the DASS-D (Table 6), K10 and K6 (Table 7) are presented. The R^2^ as a method of explaining capability and health status reveals that the condition-specific items contribute more to the estimation of capability scores/values than health status scores/values. In particular, 42.7% of total capability value can be explained through the DASS-D items, whereas the same DASS-D items explains 30.2% of variation in health status in terms of the EQ-5D-5L overall index score.

**Table 6:** Ordinary least squares regression of capability and health status on DASS-D items

	Dependent Variable			
Independent Variable	ICECAP-A	S.E.	EQ-5D-5L	S.E.
Constant	0.710	0.050	0.729	0.066
Anhedonia				
Many problems	-0.086**	0.035	-0.153***	0.045
Considerable problems	-0.030	0.028	-0.091**	0.037
Some problems	-0.016	0.023	-0.009	0.030
No problems				
Inertia				
Many problems	-0.017	0.019	-0.054**	0.025
Considerable problems				
Some problems	0.028	0.019	0.017	0.025
No problems	0.011	0.029	0.076**	0.038
Hopelessness				
Many problems				
Considerable problems	0.029	0.027	0.043	0.035
Some problems	0.074**	0.029	0.015	0.038
No problems	0.099***	0.035	0.033	0.046
Dysphoria				
Many problems	0.027	0.034	-0.056	0.045
Considerable problems	0.023	0.031	-0.079*	0.041
Some problems	0.026	0.026	-0.031	0.034
No problems				
Lack of interest/involvement				
Many problems	-0.042	0.034	-0.063	0.045
Considerable problems	-0.042	0.029	0.023	0.037
Some problems	-0.040*	0.024	0.006	0.032
No problems				
Self-depreciation				
Many problems	-0.140***	0.036	-0.092*	0.047
Considerable problems	-0.123***	0.031	-0.070*	0.041
Some problems	-0.080***	0.026	-0.061*	0.034
No problems				
Devaluation of life				
Many problems	-0.082***	0.027	-0.027	0.036
Considerable problems				
Some problems	0.001	0.023	0.015	0.030
No problems	0.019	0.029	-0.002	0.038
R^2^	0.427		0.302	
Adjusted R^2^	0.407		0.277	

S.E. standard error. *** *p*<0.01, ** *p*<0.05, *p<0.1

**Table 7:** Ordinary least squares regression of capability and health status on K10 and K6 items

	Dependent Variable							
Independent Variable	ICECAP-A	S.E.	EQ-5D-5L	S.E.	ICECAP-A	S.E.	EQ-5D-5L	S.E.
Constant	0.506	0.085	0.402	0.103	0.669	0.075	0.537	0.091
Tired								
Always	0.075**	0.036	0.019	0.044				
Mostly	0.016	0.034	-0.032	0.041				
Sometimes	0.060*	0.033	-0.003	0.040				
A little								
Never	-0.023	0.091	-0.138	0.111				
Nervous								
Always	-0.034	0.054	0.066	0.065				
Mostly	0.019	0.047	0.095*	0.058	0.061**	0.025	0.058*	0.031
Sometimes	-0.015	0.045	0.088	0.054	0.026	0.028	0.075*	0.033
A little	0.027	0.041	0.052	0.050	0.042	0.032	0.055	0.039
Never					0.017	0.045	0.011	0.055
Nervous/unease								
Always	0.011	0.053	-0.127**	0.064				
Mostly	0.057	0.042	-0.083	0.051				
Sometimes	0.058	0.035	-0.045	0.043				
A little	0.064**	0.030	-0.024	0.037				
Never								
Hopeless								
Always								
Mostly	0.067**	0.292	0.036	0.035	0.059**	0.028	0.061*	0.034
Sometimes	0.093**	0.036	-0.022	0.044	0.089**	0.035	0.019	0.042
A little	0.090**	0.042	-0.035	0.051	0.077**	0.041	0.010	0.049
Never	0.192***	0.059	0.040	0.072	0.171***	0.057	0.095	0.070
Restless/fidgety								
Always	0.034	0.051	0.014	0.062	0.081**	0.041	0.007	0.050
Mostly	-0.016	0.044	-0.016	0.053	0.030	0.037	0.000	0.045
Sometimes	-0.039	0.040	0.002	0.049	0.007	0.035	0.030	0.043
A little	-0.033	0.037	-0.007	0.045	0.002	0.034	0.016	0.042
Never								
Restless/could not sit still								
Always	0.047	0.046	-0.040	0.056				
Mostly	0.033	0.037	-0.007	0.046				
Sometimes	0.048	0.033	0.024	0.040				
A little	0.040	0.028	0.017	0.035				
Never								
Depressed								
Always								
Mostly	-0.016	0.025	0.072**	0.030				
Sometimes	0.002	0.033	0.124***	0.040				
A little	0.018	0.041	0.158***	0.050				
Never	0.113	0.085	0.216**	0.104				
Everything an effort								
Always					-0.084**	0.034	-0.147***	0.041
Mostly	0.031	0.022	0.040	0.027	-0.069**	0.031	-0.107***	0.037
Sometimes	0.075***	0.027	0.096***	0.033	-0.027	0.030	-0.032	0.036
A little	0.104***	0.037	0.109**	0.045				
Never	0.078	0.058	0.176**	0.071	-0.014	0.050	0.045	0.061
Low								
Always								
Mostly	0.036	0.028	0.112***	0.034	0.038	0.027	0.136***	0.033
Sometimes	0.036	0.033	0.103**	0.041	0.039	0.031	0.153***	0.038
A little	0.051	0.040	0.109**	0.049	0.053	0.038	0.163***	0.046
Never	-0.020	0.053	0.052	0.065	-0.030	0.049	0.120**	0.060
Worthless								
Always	-0.222***	0.053	-0.078	0.065	-0.235***	0.052	-0.105	0.064
Mostly	-0.137***	0.049	-0.073	0.059	-0.159***	0.048	-0.105*	0.058
Sometimes	-0.107**	0.045	-0.025	0.055	-0.119***	0.044	-0.053	0.054
A little	-0.043	0.041	-0.027	0.049	-0.047	0.040	-0.039	0.049
Never								
R^2^	0.372		0.343		0.340		0.316	
Adjusted R^2^	0.329		0.298		0.313		0.288	

Columns 2-5 for K10 models; Columns 6-9 for K6 models. S.E. standard error. *** *p*<0.01, ** *p*<0.05, *p<0.1

Scoring less well on the anhedonia and self-depreciation items on DASS-D appears to contribute the most to both poorer capability and health status values. Scoring lower on the hopelessness item and higher on the devaluation of life item also makes an important contribution to the capability score. The highest and lowest scores on the inertia item significantly contribute to overall health status scores.

## Discussion

This paper has attempted to assess the validity of a capability instrument for use in economic evaluation for adults with depression, using two depression-related instruments and a widely used generic health status instrument. All conceptually mapped relationships between condition-specific items and capability attributes were found to hold in statistical tests. The ICECAP-A scores were also able to differentiate individuals with depression of different severity, using clinical cut-offs from the normal to mild levels and moderate to severe levels. Further, the depression specific items appeared to more fully explain the ICECAP-A capability values than the health status values obtained through the EQ-5D-5L, as demonstrated in the multivariable regression analysis. Taken together, these analyses suggest that using capability measures provides one appropriate means of assessing depression.

This study represents the first attempt to assess the validity of using the ICECAP-A instrument specifically for individuals with depression. The data are drawn from four different nations, suggesting these results are generalisable, at least across English speaking countries. The study does have some limitations. The data collected contain only information on individuals at one point in time, so the responsiveness of the ICECAP-A instrument could not be tested here. Due to the quota sampling strategy undertaken in this study, it is unclear whether the individuals analysed here are representative of people with depression in the four countries included, but they do provide a sample with a broad range of severities of depression, which is helpful in assessing validity. This study does not conceptually map onto the EQ-5D items, as the main aim of this study was to validate the use of ICECAP-A in people with depression. The performance of EQ-5D in patients with depression and mental health more generally has been comprehensively studied previously [7]. Indeed, the key mental health themes we conceptually map the ICECAP-A items on are influenced by a study that was conducted in response to perceived inadequacy of commonly used generic health measures like EQ-5D [34]. Nonetheless, it is a limitation of this study that we did not perform a similar concept mapping exercise onto EQ-5D items.

One difficulty with a newly developed questionnaire outside traditional outcome measures, focusing on broadly defined concepts such as capability, is that there is no “gold standard” against which to assess criterion validity. Measures of capability wellbeing, like ICECAP-A, ultimately have a different normative basis to measures of health, like EQ-5D, and any decision about what measure to use to guide healthcare resource allocation must take this normative basis into account, as well as the performance of the measure. A slight concern could be raised with the similar scores seen for mild and moderate depression in adults, although there is no reason why changes in capability wellbeing should be linearly related to depression. Indeed, an alternative interpretation could be that these data indicate where the greatest impacts on capability are faced by people with depression: between no depression and mild depression; and between moderate depression and severe depression.

Further research is required on the responsiveness of the ICECAP-A measure for assessing potential benefits of new interventions for patients with depression. It would also be of benefit to assess the validity of the ICECAP-A against alternative commonly used measures of depression (e.g. Beck Depression Inventory [56]) and against other aspects of mental health. Based on the findings from the multivariable regression analysis presented here, further research into the extent to which generic capability and health status instruments provide complementary information would be useful [35, 57]. Finally, how measures of capability wellbeing, such as the ICECAP-A, can be used in economic evaluation to aid decision-making has been subject to recent research [58, 59]. Even though progress has been made with bodies like NICE now recommending capability measures for economic evaluations in social care and in the Netherlands for long-term conditions [60], more research is necessary for such measures to be used both within and beyond social care and also in applied economic evaluations. There appears to be growing recognition of the need to move beyond the current QALY approach in health economics [17], with the capability approach offering one viable alternative.

## Conclusion

The ICECAP-A measure of capability wellbeing appears to be suitable for assessing outcome in adults with depression. This study offers a number of possible policy implications. If a capability perspective were adopted in economic evaluations across a health service, this study suggests that changes in depression are more likely to be captured and valued using a generic capability instrument than the commonly used generic health status instrument, the EQ-5D (Tables 6 and 7). Provision for services preventing and treating depression, and mental health service provision more generally, is felt to play a secondary and unequal role to physical health service provision globally [61]. Using a capability perspective when assessing cost-effectiveness could potentially re-orientate resource provision across physical and mental health care services.

## Additional file

Additional file 1: Concept Mapping Methods. This file contains the detailed approach taken in developing the conceptual map. (DOCX 26 kb)

## Abbreviations

DALYs: Disability adjusted life years
DASS-21: Depression, anxiety and stress scale 21 item
DASS-D: Depression subscale of DASS-21
EQ-5D-5L: EuroQol five dimension five level
HRQoL: Health related quality of life
ICECAP: Investigating Choice Experiments CAPability suite of measure
ICECAP-A: Measure of capability wellbeing for Adults (18+ years old)
ICECAP-O: Measure of capability wellbeing for older people (65+ years old)
ICECAP-SCM: Supportive care measure developed for palliative care
K10: Kessler psychological distress scale 10 item
K6: Kessler psychological distress scale 6 item
MIC: Multi Instrument comparison dataset
NICE: National Institute for Health and Care Excellence
OLS: Ordinary Least Squares
QALYs: Quality adjusted life years
