# Supplementary material for: Assessing the validity of the ICECAP-A capability measure for adults with depression
Source: BMC Psychiatry. 2017 Feb 2;17:46. doi: 10.1186/s12888-017-1211-8 (PMC5289054; doi:10.1186/s12888-017-1211-8)
Supplement: Additional file 1: — Concept Mapping Methods. This file contains the detailed approach taken in developing the conceptual map. (DOCX 26 kb) [file 12888_2017_1211_MOESM1_ESM.docx]

# Additional File 1.

# Concept mapping methods

In this study, a recent synthesis of qualitative research that developed quality of life domains for people with mental health problems, is used to determine expected relationships between instruments measuring capability (ICECAP-A), condition-specific depression (DASS-D) and mental health problems (K10). In Table 1, the list of Connell et al. (2012) quality of life themes for people with mental health problems is presented, as well as detailed attributes for each of the domains.

**Table 1. Quality of life of people with mental health problems by Connell et al. (2012)**

| **Well-Being/**  **Ill-Being** | **Control/**  **Autonomy/**  **Choice** | **Self Perception** | **Belonging** | **Activity/**  **Employment** | **Hope and Hopefulness** |
| --- | --- | --- | --- | --- | --- |
| Distress/  Subjective experience of symptoms | Control (general) | Self-identity/  sense of self | Belonging/  Being part of the community | Activity general | Hope/  hopelessness |
| Experience of psychosis/  mania | Symptom control/  management | Self-efficacy | Good relationships | Employment | Goals/  personal achievement |
| Fear/  Anxiety/  Worry | Information and understanding of illness | Self-esteem | Support | Meaningful/ enjoyable/  suited to needs | Loss and effect of past experiences |
| Energy/  motivation | Choice (general) | Self-acceptance/  self-stigma | Acceptance/ understanding | Routine and structure |  |
| Well-being (positive concepts eg enjoyment/  relaxation/  stability) | Choice – related to limited finances |  | Company/  camaraderie/  shared interests |  |  |
| Physical Well-being | Choice – related to job opportunities |  | Love, care and affection |  |  |
|  | Independence/  dependence |  | Difficulties forming and maintaining relationships |  |  |
|  | Personal strength, determination, self sufficiency |  | Stigma |  |  |
|  |  |  | Feeling normal |  |  |
|  |  |  | Loneliness/  Isolation, alienation |  |  |

Each of the six quality of life theme from Table 1 is conceptually mapped to the 5 items on the ICECAP-A capability instrument (Table 2) and the 7 items on DASS-D (depression scale – Table 3) and K10 (mental health scale – Table 4). This means that for a relationship to exist between the self perception mental health quality of life theme and the autonomy item on the ICECAP-A, a relationship would be expected if there is cross-over between **any** of the attributes attached to each theme and item.

**Table 2. ICECAP-A items and attributes**

| **Stability** | **Attachment** | **Autonomy** | **Achievement** | **Enjoyment** |
| --- | --- | --- | --- | --- |
| continuity of friends | love | look after oneself | Move forward in life | Quiet pleasures |
| continuity of work | support | Independence in decision-making | Attain goals | fun |
| Continuity of location | social contact | privacy | pride | exciting |
|  |  | identity | Recognition and appreciation |  |

**Table 3. DASS-D items and descriptions**

| **anhedonia** | **inertia** | **hopelessness** | **dysphoria** | **Lack of interest/**  **involvement** | **Self-depreciation** | **Devaluation of life** |
| --- | --- | --- | --- | --- | --- | --- |
| Unable to experience enjoyment and satisfaction | Slow, lacking in initiative | Pessimistic about the future | Dispirited, gloomy and blue | Unable to become interested or involved | Being self-disparaging | Convenience of life has no meaning |

**Table 4. K10 items**

| **Question** | **K6** |
| --- | --- |
| K1. Tired for no good reason |  |
| K2. Feel nervous | √ |
| K3. Nervous so that nothing can calm you down |  |
| K4. Hopeless | √ |
| K5. Restless or figidity | √ |
| K6. Restless so that you could not sit still |  |
| K7. Feel depressed |  |
| K8. Everything was an effort | √ |
| K9. So sad that nothing could cheer you up | √ |
| K10. Worthless | √ |

To conceptually map between instruments, the first step required a relationship between the 6 mental health domains and each instrument respectively to be established. The result of these expected relationships are presented in table 5 (for ICECAP-A), table 6 (for DASS-D) and table 7 (for K10).

**Table 5. ICECAP-A items conceptually mapped onto mental health quality of life domains**

|  | **Stability** | **Attachment** | **Autonomy** | **Achievement** | **Enjoyment** |
| --- | --- | --- | --- | --- | --- |
| Well-Being/  Ill-Being | √ | √ | √ | √ | √ |
| Control/  Autonomy/  Choice | √ |  | √ | √ |  |
| Self Perception |  |  | √ | √ |  |
| Belonging | √ | √ | √ | √ |  |
| Activity/  Employment | √ |  |  | √ | √ |
| Hope/  Hopelessness |  |  |  | √ |  |

**Table 6. DASS-D items conceptually mapped onto mental health quality of life domains**

|  | anhedonia | inertia | hopelessness | dysphoria | Lack of interest/  involvement | Self-depreciation | Devaluation of life |
| --- | --- | --- | --- | --- | --- | --- | --- |
| Well-Being/  Ill-Being | √ | √ | √ | √ | √ | √ | √ |
| Control/  Autonomy/  Choice | √ | √ | √ | √ |  |  |  |
| Self Perception |  | √ | √ | √ |  | √ | √ |
| Belonging | √ |  | √ | √ | √ | √ | √ |
| Activity/  Employment | √ | √ | √ | √ | √ |  |  |
| Hope/  Hopelessness | √ |  | √ |  |  |  | √ |

**Table 7. K10 items conceptually mapped onto mental health quality of life domains**

|  | K1 | K2 | K3 | K4 | K5 | K6 | K7 | K8 | K9 | K10 |
| --- | --- | --- | --- | --- | --- | --- | --- | --- | --- | --- |
| Well-Being/  Ill-Being | √ | √ | √ | √ | √ | √ | √ | √ | √ | √ |
| Control/  Autonomy/  Choice | √ |  |  | √ |  |  | √ | √ | √ |  |
| Self Perception |  | √ | √ | √ |  |  | √ | √ |  | √ |
| Belonging |  |  |  | √ |  |  | √ |  | √ | √ |
| Activity/  Employment |  |  |  | √ |  |  | √ | √ | √ |  |
| Hope/  Hopelessness |  |  |  | √ |  |  | √ |  |  |  |

In order to assess whether relationships are expected between items on instruments, the results of the first step of the conceptual mapping process are used as a basis to compare quality of life themes in people with mental health problems for similarities across instruments. For an expected relationship between two items on each instrument to occur, it is expected that each item will have at least as many of the quality of life domains in common with each other. So if two items (say one item on ICECAP-A and one item on DASS-D) have 2 out of 4 themes in common, we would expect a relationship between those two items (≥50%), while if two items have 1 out of 4 domains, a relationship between those two items is not expected (<50%). The result of this process is presented in Table 8 for ICECAP-A and DASS-D items and Table 9 for ICECAP-A and K10 items.

**Table 8. Concept mapping DASS-D and ICECAP-A item relationships by mental health themes (%)**

|  | Stability | Attachment | Autonomy | Achievement | Enjoyment |
| --- | --- | --- | --- | --- | --- |
| Anhedonia | **0.89** | **0.57** | **0.67** | **0.91** | **0.57** |
| Inertia | **0.75** | 0.33 | **0.75** | **0.80** | **0.67** |
| Hopelessness | **0.80** | **0.50** | **0.80** | **1.00** | **0.50** |
| Dysphoria | **0.89** | **0.57** | **0.89** | **0.91** | **0.57** |
| Lack of interest/  involvement | **0.86** | **0.80** | **0.57** | **0.67** | **0.80** |
| Self-depreciation | **0.57** | **0.80** | **0.88** | **0.67** | 0.40 |
| Devaluation of life | **0.50** | **0.67** | **0.75** | **0.80** | 0.33 |

Relationships in bold where items between two measures have at least half of Connell’s mental health themes in common

**Table 9. Concept mapping K10 and ICECAP-A relationships by mental health themes (%)**

|  | Stability | Attachment | Autonomy | Achievement | Enjoyment |
| --- | --- | --- | --- | --- | --- |
| K1 | **0.67** | **0.50** | **0.67** | **0.50** | **0.50** |
| K2 | 0.33 | **0.50** | **0.67** | **0.50** | **0.50** |
| K3 | 0.33 | **0.50** | **0.67** | **0.50** | **0.50** |
| K4 | **0.80** | **0.50** | **0.80** | **1.00** | **0.50** |
| K5 | 0.40 | **0.67** | 0.40 | 0.29 | **0.67** |
| K6 | 0.40 | **0.67** | 0.40 | 0.29 | **0.67** |
| K7 | **0.80** | **0.50** | **0.80** | **1.00** | **0.50** |
| K8 | **0.75** | 0.40 | **0.75** | **0.80** | **0.67** |
| K9 | **1.00** | **0.67** | **0.75** | **0.80** | **0.67** |
| K10 | **0.57** | **0.80** | **0.86** | **0.67** | 0.40 |

K10, Kessler Psychological Distress Scale 10 item; k1, tired for no good reason; k2, feel nervous; k3, nervous so that nothing could calm you down; k4, hopeless; k5, restless or fidgety; k6, restless that you could sit still; k7, feel depressed; k8, everything was an effort; k9, so sad that nothing could cheer you up; k10, worthless.

Relationships in bold where items between two measures have at least half of Connell’s mental health themes in common

The relationships highlighted in bold are used to develop concept maps between items on the capability instrument (ICECAP-A) with depression-specific items on DASS-21 (DASS-D scale). These concept maps are presented in Figure form in the full article (Figure 1).
